# Genome-Wide Identification of UGT Gene Family and Functional Analysis of *PgUGT29* in *Platycodon grandiflorus*

**DOI:** 10.3390/ijms26104832

**Published:** 2025-05-18

**Authors:** Jizhou Fan, Weiyi Rao, Daiyin Peng, Tao Wei, Shihai Xing

**Affiliations:** 1 College of Pharmacy, Anhui University of Chinese Medicine, Hefei 230012, China; 17805652827@stu.ahtcm.edu.cn (J.F.); r13694358rwy@163.com (W.R.); pengdy@ahtcm.edu.cn (D.P.); 2 MOE-Anhui Joint Collaborative Innovation Center for Quality Improvement of Anhui Genuine Chinese Medicinal Materials, Hefei 230038, China; 3 College of Integrated Chinese and Western Medicine, Anhui University of Chinese Medicine, Hefei 230012, China; 4 Joint Research Center for Chinese Herbal Medicine of Anhui of IHM, Anhui University of Chinese Medicine, Hefei 230012, China; 5 Anhui Province Key Laboratory of Research & Development of Chinese Medicine, Hefei 230012, China

**Keywords:** *Platycodon grandiflorus*, UGT, triterpenoid saponins, functional characterisation

## Abstract

Uridine diphosphate glycosyltransferase (UGT) is a core protein for glycosylation of plant natural products and other small molecules. Although many studies on functional identification of UGTs are now available, analysis of UGTs in *Platycodon grandiflorus* is still relatively scarce. We identified 107 PgUGTs genome-wide from *P. grandiflorus* and investigated their phylogenetic relationships, chromosomal localisation, collinearity, cis-regulatory elements, motifs, domains, and gene structures. PgUGT29 and PgUGT72 were two putative glycosyltransferases for platycodins biosynthesis in *P. grandiflorus* according to our previous study and bioinfornatical analyses. In vitro enzyme activity showed that PgUGT29 can catalyse the glycosylation of the C3 position of Platycodin D (PD) to generate Platycodin D3 (PD3), while candidate enzyme PgUGT72 does not function as a glycosyltransferase. Molecular docking indicated that T145, D392, Q393, and N396 may be the crucial residues for PgUGT29 to catalyse the generation of PD3 from UDP-Glc and PD. In this study, we identified and cloned PgUGT29, elucidated its catalytic function in converting PD to PD3, and predicted key residues critical for its enzymatic activity. These findings provide a theoretical foundation and technical framework for future targeted metabolic engineering and directional regulation of medicinal components in *Platycodon grandiflorus*.

## 1. Introduction

*Platycodon grandiflorus (Jacq.) A. DC.*, which is often used medicinally and consumed, is a perennial herb of the genus Platycodon in the family Campanulaceae with various pharmacological activities, such as anti-inflammatory, anti-tumour, anti-obesity, and cognitive enhancement [1,2]. The major important active ingredients of *P. grandiflorus* are Platycodin D (PD), Platycodin D3 (PD3), and Platycoside E, all of which belong to the oleanane-type pentacyclic triterpene saponins. The oleanane-type pentacyclic triterpenoid saponins in *P. grandiflorus* are usually di-glycosylated saponins, and they are mainly attached to glycosyl at the C-3 and C-28 positions. The types of glycosyl groups attached mainly include D-glucose, L-arabinose, L-rhamnose, D-xylose, D-apiose, and their derivatives [1,3]. The saponins of *P. grandiflorus* can be divided into three kinds of saponins according to their mother nucleus: polygalacic acid, platycogenic acid, and platycodic acid; the most active, Platycodin D, belongs to platycodic acid [4,5]. Currently, the platycodic acid saponins isolated from *P. grandiflorus* include Platycodigenin, Platycodin F, PD, Deapi-platycodin D, platycoside E, Deapi-platycoside E, Platycodin D2, and PD3 (Supplementary Figure S1A) [3]. The pharmacological and biological activities of the saponins are mainly associated with the number and type of the glycosyl side chains.

The formation of different triterpenoid saponins requires a process of synthesis and modification of the triterpenoid saponin backbone [6,7]. Whereas the inter-conversion between triterpenoid saponins is in the process of glycosylation modification, the addition of glycosyl groups to the glycosyl chain is called glycosylation, and vice versa, it is called de-glycosylation. Glycosyltransferases (GTs) are a class of enzymes that catalyse glycosylation reactions by transferring glycosyl from activated donor molecules to acceptor molecules to produce a wide variety of glycosides [8,9,10]. Among them, glycosyltransferase family 1 (GT1) uses uridine diphosphate glycosyl as the glycosyl donor, so it is often called uridine diphosphate glycosyltransferase (UGT). The function of many UGTs in oleanane-type pentacyclic triterpenoid saponins has now been reported. Meesapyodsuk et al. [11] identified and cloned UGT74M1 from *Saponaria vaccaria* and found that it is highly expressed in roots and leaves and catalyses the C28-COOH glycosylation of gypsogenic acid to produce the corresponding monosaccharide glycoside. Tang et al. [12] identified four glycosyltransferases, PzGAT1, PzGAT2, PzGAT3, and PjGAT1, all of which catalyse the C3-OH glucuronidation of oleanolic acid to produce calenduloside E from *Panax zingiberensis* and *Panax japonicus*, respectively. Zhang et al. [13] identified two UGTs (PgUGT18 and PgUGT8) from *Panax ginseng* that catalyse the glycosylation of the second glycosyl moiety of C3 and C28-COOH, respectively, in the biosynthetic pathway of oleanane-type ginsenosides. In vitro enzyme activity experiments showed that PgUGT18 catalysed the extension of the C3 glycosyl chain of calenduloside E and chikusetsusaponin Iva to produce zingibroside R1 and ginsenoside Ro, respectively. PgUGT8 catalysed C28-COOH glycosylation of oleanolic acid, calenduloside E, and zingibroside R1 to generate olanolic acid 28-O-glucopyranosyl ester, chikusetsusaponin IVa, and ginsenoside Ro. Then, they integrated GTs in combinations into *Saccharomyces cerevisiae* genome and realised de novo biosynthesis of oleanane-type ginsenosides with a yield of 1.41 μg/L ginsenoside Ro in shake flasks. Tang et al. [14] investigated two UGTs (PjmUGT1 and PjmUGT2) in *Panax japonicus*. Through bioinformatics analysis, heterologous expression, and enzyme activity characterisation, it was found that PjmUGT1 could catalyse the C28-COOH glycosylation of calenduloside E and zingibroside R1 to generate chikusetsuponin IVa and ginsenoside Ro. PjmUGT2 prolonged the C3-glycosyl chains of calenduloside E and chikusetsusaponin IVa to generate zingibroside R1 and ginsenoside Ro, respectively.

For the synthesis of triterpene saponins from *P. grandiflorus*, in our previous study, we found that Pgβ-glucosidase could convert PE to PD [15]. After that, Tang et al. [16] also characterised PgGT1 by matrix-assisted laser desorption/ionisation mass spectrometry imaging (MALDI MSI)-assisted genome mining strategy. PgGT1 can convert PD into PD3 and then further into PE. However, there are still fewer studies on the effect of PgUGT on catalysing *P. grandiflorus* saponins synthesis. Therefore, we hypothesised a biosynthetic pathway from platycodigenin to PE [16,17,18,19] (Supplementary Figure S1B).

The purpose of this paper is to analyse the UGT gene family of *P. grandiflorus* and unite the gene expression pattern and saponin content distribution in its different organisations. We selected and expressed PgUGT, which may have a catalytic effect on the C-3 position of the *P. grandiflorus* saponin backbone, and verified and elucidated its function by in vitro enzyme activity experiments and molecular docking. Our findings may provide new perspectives on the synthesis pathway of *P. grandiflorus* saponins.

## 2. Results

### 2.1. Sequence Acquisition and Physicochemical Characterisation of PgUGT Proteins

A total of 113 protein sequences were obtained by comparison with the HMM model file PF00201 in the *P. grandiflorus* protein database with the E-value set to e^−10^. The C-terminus of all plant UGTs contains a highly conserved sequence covering 44 contiguous amino acids, known as the PSPG structural domain [20,21]. UGTs recognise and bind UDP-glycosyl by this sequence. In contrast, the N-terminal structural domain is loosely bound to glycosides, which can lead to a diversity of substrate structures. The PSPG-conserved motifs of PgUGTs were found and analysed in Bioedit V.7.7.1.0, and sequences without conserved motifs were deleted to obtain the final 107 PgUGT sequences, named PgUGT1 to PgUGT107 (Supplementary Table S1).

The molecular weight, isoelectric point, GRAVY, and other information of the obtained PgUGT proteins were analysed using the ProtParam online tool. The results reveal that 107 PgUGT proteins had lengths ranging from 131 to 923 amino acids, relative molecular masses ranging from 14.62 to 103.58 kDa, with an average relative molecular mass of 50.48 kDa, and isoelectric points ranging from 4.72 to 8.77, with an average isoelectric point of 5.71. Fourteen of the PgUGTs (PgUGT32, PgUGT101, PgUGT71, PgUGT39, PgUGT35, PgUGT60, PgUGT106, PgUGT105, PgUGT31, PgUGT44, PgUGT94, PgUGT82, PgUGT47, and PgUGT97) had a positive GRAVY, and the rest had a negative GRAVY, suggesting that the majority of PgUGT proteins are hydrophilic. Prediction of the subcellular localisation of PgUGTs showed that PgUGTs were located in the cytoplasm (51.40%), chloroplasts (38.32%), nucleus (9.35%), and extracellular interstitial space (0.93%), respectively (Supplementary Table S2).

### 2.2. Phylogenetic Tree Analysis of PgUGT Proteins

**Figure 1:**
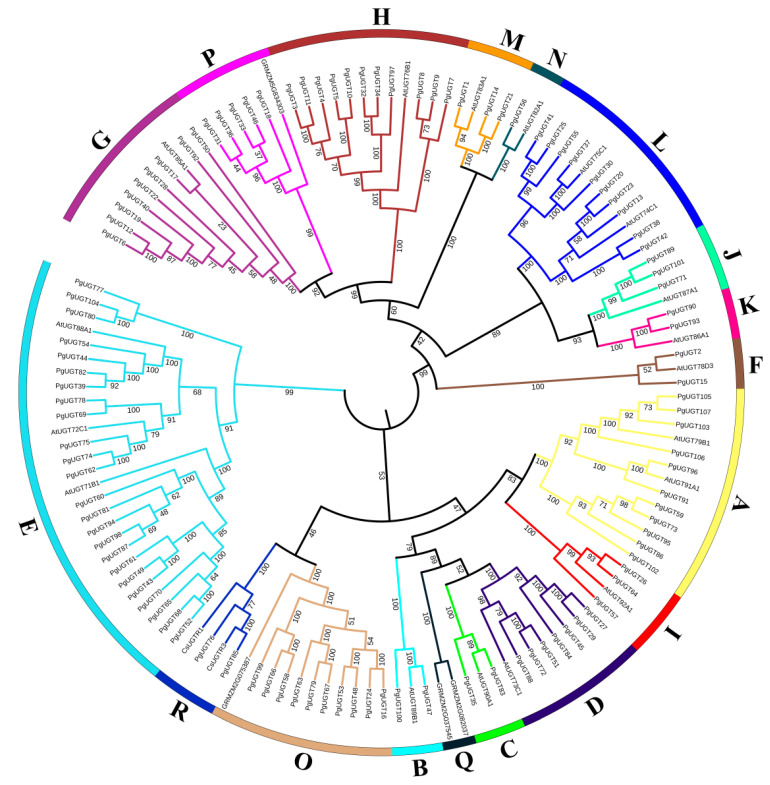
Phylogenetic tree of PgUGTs in *P. grandiflorus*. The phylogenetic tree was constructed by using the full-length sequences of 107 PgUGTs and 24 UGTs from *A*. *thaliana*, *Z*. *mays*, and *C*. *sinensis*. The 18 groups (A–R) are indicated in different colours.

We conducted phylogenetic tree analysis by combining the PgUGTs with 18 *A. thaliana* UGTs, 4 *Z. mays* UGTs, and 2 *C. sinensis* UGTs, and thus classified the 107 PgUGTs into 17 groups (Figure 1). Bootstrap values for the corresponding nodes are all greater than 95 with high confidence. Phylogenetic analysis showed that these seventeen groups were groups A-N already identified in *A. thaliana*, groups O and P in *Z. mays*, and group R in *C. sinensis*, and that there was no PgUGT in group Q. Twelve UGTs in ginseng were selected by Khorolragchaa et al. as the most probable candidates for the synthesis of triterpenoids, belonging to six groups (A, E, G, L, N, and O) [22]. Most of the PgUGTs were also clustered in groups E (24), A (11), H (11), L (10), O (10), and G (9). This suggests that most of PgUGTs were also likely to be associated with the synthesis of triterpenoids.

### 2.3. Chromosomal Localisation and Collinearity Analyses of PgUGTs

**Figure 2:**
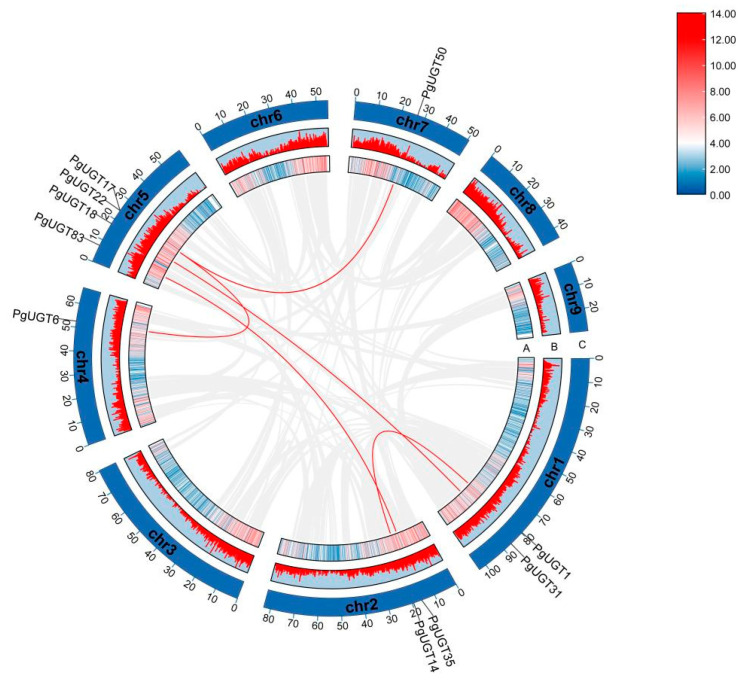
The duplicated event is a collinearity block in the *P. grandiflorus* genome, and the segmental duplication of PgUGTs are drawn with red lines. (**A**) Line colours indicate gene density. (**B**) The red line represents gene density. (**C**) Chr1-Chr9 represents the 9 chromosomes of *P. grandiflorus*.

In order to study the distribution of all PgUGT genes on chromosomes in general, the localisation of UGT genes on chromosomes was further investigated based on our available genomic information of *P. grandiflorus* (Supplementary Figure S2). Among them, 106 UGT genes were generally distributed on nine chromosomes, and 1 UGT gene was localised to contigs. Specifically, UGT genes were preferentially localised on Chr1 (23 UGTs), followed by Chr3 (22 UGTs), and Chr9 had the lowest number of UGT genes, with only three. The results of the analyses showed that a total of 32 tandem duplication gene pairs and 5 fragment duplication gene pairs were identified in 107 PgUGTs. The tandem duplication events are more frequent in Chr1 and Chr3, and the fragment duplication events are more frequent in Chr5 (Figure 2).

### 2.4. Cis-Regulatory Elements in PgUGT Promoters

**Figure 3:**
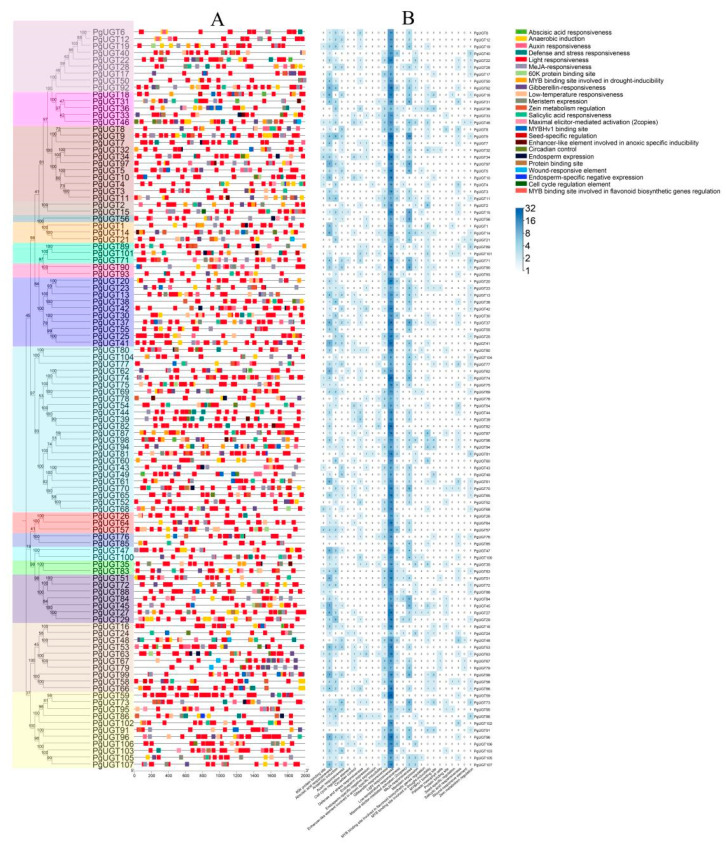
Analysis of cis-regulatory elements in PgUGT promoters. (**A**) Cis-acting elements of 2 kb promoter regions of PgUGT genes. (**B**) Heatmap analysis of Cis-regulatory elements of 2 kb promoter regions of PgUGT genes.

To further investigate the PgUGT genes, we used PlantCare to extract cis-regulatory elements from the 2 kb region 5′ upstream of the PgUGT genes. As Figure 3 shows, we predicted a total of 25 cis-regulatory elements in the PgUGT genes. Among them, the most numerous were Light, MeJAs, Abscisic acid, Gibberellin, Auxin responsiveness, Low-temperature responsiveness, and Anaerobic induction. Light responsiveness was the most frequent element, suggesting that the action of UGT on saponins may be regulated by light.

### 2.5. Conserved Motifs, Domains, and Gene Structures of PgUGTs

Through the Meme online website, 10 motifs were obtained from PgUGTs with widths ranging from 7 to 43aa, where Motif 1 included the PSPG motifs of UGT (Supplementary Figure S3A,D). Motif analysis showed that the 10 motifs were conserved in most groups. However, Motif 5 was absent in groups A and R, Motif 2 was absent in group C, Motifs 8 and 10 were absent in group F, Motifs 5, 6, 9, and 10 were absent in group N, and Motifs 6 and 10 were absent in group J. Motif 5 was absent in the vast majority of UGTs in group E. The different Motif compositions may lead to diversity in gene function.

Domain analysis showed five conserved structural domains in 107 PgUGTs, of which all 107 PgUGTs had the domains GT1_Gtf-like, PLN02448, and Glycosyltransferase_GTB-type Superfamily (Supplementary Figure S3B). All three domains belong to GT-B folding, which indicates that all 107 PgUGT proteins are GT-B folding. This indicates that these 107 PgUGTs share a common GTB topology, which is one of the two protein topologies observed for nucleotide-glycosyl-dependent glycosyltransferases. In contrast, the GED and PUF573 domains, which PgUGT83 possesses, suggest that PgUGT83 may function differently from other PgUGTs.

To reveal the structural evolution of the PgUGT gene family, the exon–intron structures of the PgUGT genes were analysed (Supplementary Figure S3C). The results show that the number of exons in PgUGTs varies from one to seven, but the vast majority of PgUGTs contain only one or two exons. Among them, PgUGT36 has the most at seven exons. Of the 107 PgUGTs, 55 (51%) PgUGTs contained two UTRs, and 30 (28%) PgUGTs were missing UTRs.

### 2.6. Screening of Candidate PgUGTs

To further investigate the catalytic effect of PgUGTs on *P. grandiflorus* saponins, 24 GTs with catalytic effects on the C3 position of the saponin backbone were used to perform blast comparisons with 107 PgUGTs in NCBI, and finally, heatmaps were plotted with bit scores (Supplementary Figure S4).

**Figure 4:**
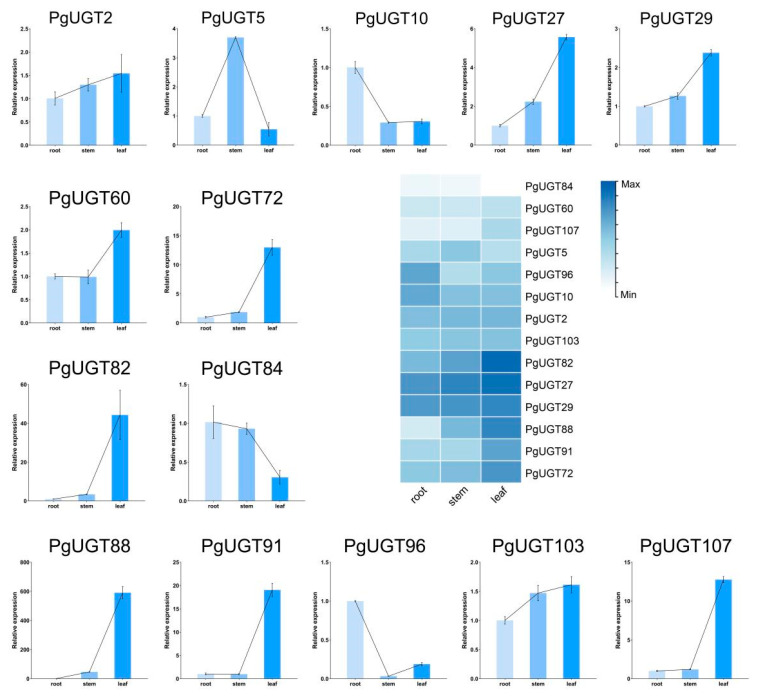
Expression of 14 *PgUGTs* genes in roots, stems, and leaves. The heatmap in the middle shows the expression pattern of 14 PgUGTs in root, stem, and leaf.

As a result, it was found that PgUGTs with high similarity to 24 UGTs with identified functions were clustered in groups L, D, and A. BvUGT1, PgUGT18, PjmUGT2, UGT73C10, UGT73C11, UGT73C12, UGT73C13, UGT73C21, UGT73C22, UGT73C23, UGT73C25, UGT73C26, and UGT73C27, which are highly similar to the PgUGTs of group D, have a catalysing effect on the C3 position of the oleanane-type saponins [13,14,23,24]. In addition, PgGT1 and UGT91H9 have similar functions [16,25]. Thus, we selected PgUGT27, PgUGT29, PgUGT84, PgUGT72, PgUGT88, PgUGT96, and PgUGT91, which had a high degree of similarity to them, as candidate genes for catalysing the binding of zeylanolide saponin C3 position to glucose. Subsequently, seven PgUGTs from groups H, F, E, and A, respectively, whose members exist in other species and were verified to transfer arabinose glycosyl and rhamnose glycosyl, were selected [26,27,28]. RT-qPCR was performed on these 14 PgUGTs to study their expression patterns in the roots, stems, and leaves of *P. grandiflorus* (Figure 4). The results show that PgUGT5 and PgUGT10 in group H had high expression in stems and roots. PgUGT2 in group F had slightly higher expression in leaves than in stems and slightly higher expression in stems than in roots, but the difference in expression in roots, stems, and leaves was not significant. PgUGT82 and PgUGT60 in group E did not have any significant difference in expression in roots and stems but had significantly higher expression in leaves than in roots and stems. PgUGT27, PgUGT29, PgUGT72, and PgUGT88 in group D showed consistent expression patterns, with significantly higher expression in leaves than in stems and significantly higher expression in stems than in roots, whereas the expression of PgUGT84 in leaves was significantly lower than in roots and stems. In group A, PgUGT91, PgUGT103, and PgUGT107 were significantly higher in leaves than in roots, whereas PgUGT96 was highly expressed in roots. We found that different groups of PgUGTs showed the same expression trend, and among PgUGT27, PgUGT29, PgUGT84, PgUGT72, PgUGT88, PgUGT96, and PgUGT91 as candidate genes, the expression of the five genes in leaves was significantly higher than that in roots and stems except for PgUGT84 and PgUGT96. The high expression of PgUGT in leaves is also consistent with the results of the analysis of cis-regulatory elements, which suggest that PgUGT may respond to light and thus function accordingly in leaves.

In our previous study, we found that PD was slightly lower in leaves than in roots and stems, but these differences were not significant, and PE was significantly higher in leaves than in roots and stems. It is obvious that the gene expression patterns of the candidate PgUGTs are synergistic with the distribution of the contents of PD and PE, and the parts of *P. grandiflorus* with high expression of the candidate PgUGTs have correspondingly high contents of PE. Thus, it can be speculated that these genes can transfer glucose from UDP-glc to the C3 position of PD and then generate PE. In the subsequent experiments, we randomly selected PgUGT29 and PgUGT72 among them for protein expression and purification and then verified their functions.

### 2.7. Protein Expression and Purification

**Figure 5:**
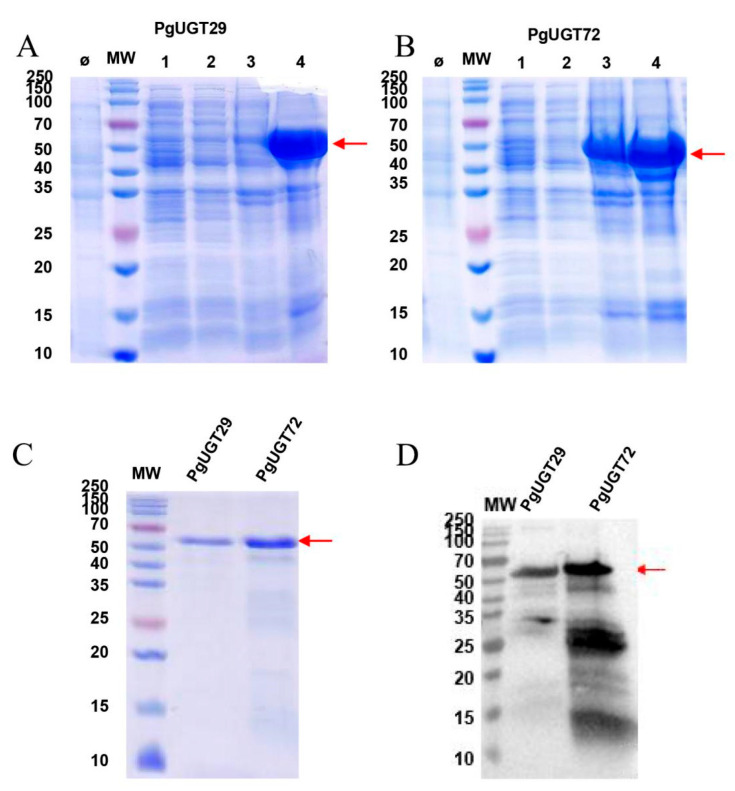
SDS-PAGE and Western blot analysis of the recombinant protein. MW: protein molecular weight standards; Ø: uninduced strains. (**A**) SDS-PAGE analysis. Line 4: crude extract of PgUGT29. (**B**) SDS-PAGE analysis. Line 4: crude extract of PgUGT72. (**C**) SDS-PAGE analysis of purified PgUGT29 and PgUGT72 protein. (**D**) Western blot analysis of purified PgUGT29 and PgUGT72 protein.

After recombinant protein construction, the expected PgUGT29 and PgUGT72 proteins were 56.57 kDa and 57.32 kDa, respectively. The SDS-PAGE results show that the target proteins were well expressed when induced and cultured for 4h at 37 °C, 1 mM IPTG condition, using uninduced strain as a negative control (Figure 5A,B). After protein purification, SDS-PAGE and WB were performed again (Figure 5C,D). The final results show that the PgUGT29 and PgUGT72 proteins were successfully expressed, and the measured final concentrations were 0.21 mg/mL and 0.50 mg/mL, respectively.

### 2.8. Functional Validation and Molecular Docking

**Figure 6:**
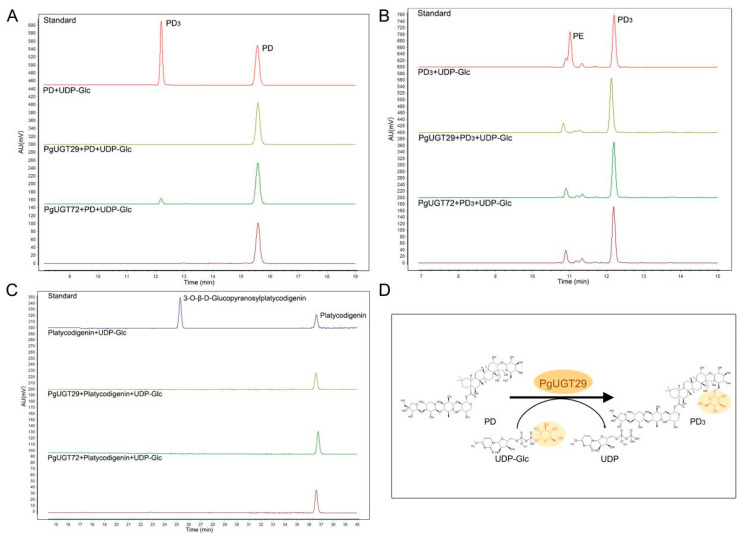
In vitro assays of enzymatic activity of PgUGT29 and PgUGT72. (**A**) HPLC analysis of in vitro reaction products with PD as substrate, (**B**) HPLC analysis of in vitro reaction products with PD3 as substrate, and (**C**) HPLC analysis of in vitro reaction products with platycodigenin as substrate. (**D**) PgUGT29 catalyses the glycosylation of PD to produce PD3.

To further determine the functions of PgUGT29 and PgUGT72, we took HPLC measurements of the enzyme reaction products (Figure 6). The results show that PgUGT29 could catalyse the combination of PD and Glc to produce PD3 when PD was the substrate and UDP-Glc was the donor, whereas PgUGT72 did not have the same function (Figure 6A). Neither PgUGT29 nor PgUGT72 could catalyse the generation of PE from PD3 when PD3 was used as a substrate and UDP-Glc as a donor (Figure 6B). Neither PgUGT29 nor PgUGT72 could catalyse the generation of 3-O-β-D-Glucopyranosylplatycodigenin from platycodigenin when UDP-Glc was the substrate and UDP-Glc was the donor (Figure 6C).

**Figure 7:**
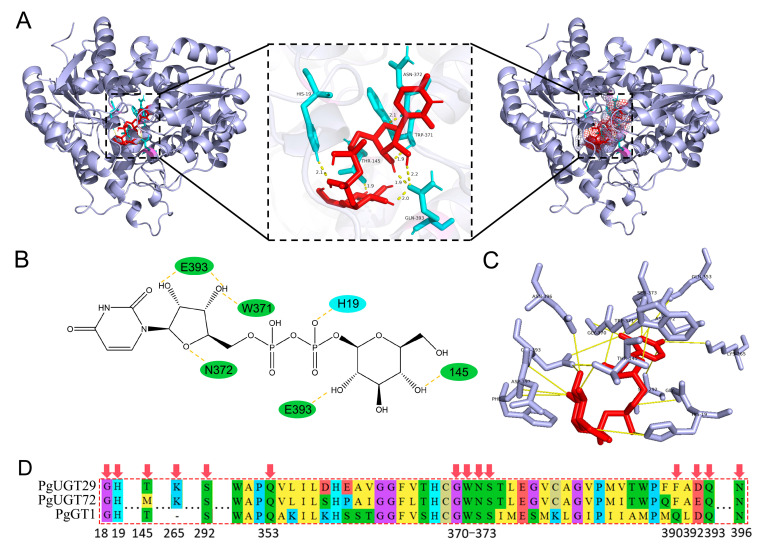
Homology modelling of PgUGT29 and its docking with UDP-Glc. (**A**) Molecular docking of PgGT29/UDP-Glc. The key residues and substrate molecules are shown as sticks. Hydrogen bonds are indicated by yellow broken lines. (**B**) Two-dimensional structure of Autodock molecular docking results. (**C**) Hydrogen bonding in the 5.0 Å range of the substrates. (**D**) The conserved protein sequence of the PSPG box in PgUGT29, PgUGT72, and PgGT1. The key residues mentioned above are marked with red arrows.

In order to clarify the molecular basis for the binding of PgGT29 and UDP-Glc, molecular docking of PgGT29 and UDP-Glc was performed to identify crucial amino acid residues. In general, we consider that the docking results are feasible for binding energies less than −1.2 kcal/mol or less than −5 kj/mol. The lowest energy conformational binding energy of UDP-Glc and PgUGT29 was −6.25 kcal/mol, which indicates that our docking results are stable and reliable. As shown in Figure 7A, UDP-Glc was completely encapsulated by PgUGT29 in a narrow channel. There, UDP-Glc interacts with the PSPG motif at the C-terminus of PgUGT29 (Figure 7A,B). The docking results show that a total of seven hydrogen bonds were generated between UDP-Glc and PgUGT29, including five hydrogen bonds formed between UDP-Glc and amino acids on the PSPG motif. W371 forms a hydrogen bond with a hydroxyl oxygen atom close to the phosphate residue on a ribose, and N372 forms a hydrogen bond with an oxygen atom on the backbone of the ribose. Q393 can form a hydrogen bond with the two hydroxyl hydrogen atoms on the ribose and with a hydroxyl hydrogen atom at the C2 position of glucose, respectively. In addition, H19 can form a hydrogen bond with an oxygen atom close to the phosphate residue of glucose, and T145 can form a hydrogen bond with the hydroxyl hydrogen atom at the C4 position of glucose. It has been shown that UGTs recognise and bind UDP-glycosyl via the C-terminal PSPG motif, whereas the N-terminal structural domains are loosely bound to glycosides, which can lead to a diversity of substrate structures. This implies that PgUGT29 may bind the glucose portion of UDP-Glc by recognition of Q393 in the PSPG motif. PgUGT29, PgUGT72, and PgGT1 were aligned in multiple sequence comparisons to find the PSPG motif.

In order to further investigate the binding mechanism of PgUGT29 and UGT-Glc, we further analysed Autock’s results by expanding the hydrogen bonding distance to a range of 5 Å at the PLIP online website and found that F390,D392 also formed hydrogen bonds with the hydroxyl hydrogen atom at the C2 position of glucose (Figure 7C). D392 is an E residue at the corresponding position of PgUGT72. This suggests that the recognition of glucose residues by PgUGT may not be singularly dependent on Q393 but may be determined by relying on consecutive DQ or ADQ residues. Two consecutive DQ residues are at the end position of the PSPG sequence of PgGT1 (Figure 7D). In addition, Q393 and N396 also form hydrogen bonds with the hydroxyl group at the C3 position of glucose. The same N residue appears in PgGT1 at the position corresponding to N396 of PgUGT29. These results suggest that the recognition and binding of UGT-Glc by PgUGT29 may be related to T145, D392, Q393, and N396, and that residues D392 and Q393 may be joint coactivators.

## 3. Discussion

In this study, we first identified 107 PgUGTs from the *P. grandiflorus* genome and investigated their physicochemical properties. The PgUGTs were classified into 17 groups based on their phylogenetic relationships with UGTs from other species. There have been many studies on the classification of UGT families. Barvkar et al. [29] clustered and analysed the protein sequences of typical Arabidopsis thalian UGTs and Linum Usitatisimum UGTs and classified them into 14 major subfamilies (A–N). An evolutionary tree was constructed by aligning the sequences of the identified Zea mays UGTs, 18 UGTs (A–N) from A. thaliana and 2 from Oryza sativa, into 17 groups (A–Q). This was accomplished by performing sequence comparison, thus classifying the UGTs of *Z. mays* into 17 groups (A–Q), of which groups O, P, and Q are new groups found in *Z. mays* [30]. Cui et al. [31] identified 132 UGTs in *Camellia sinensis*, and the cluster analysis showed that they were distributed within 16 groups, which were A–M, O, P, and a new group R. There was no PgUGT in group Q. This indicated that there were dissimilarities of UGTs among different species, and with the deepening and expanding of the study, the UGT family was constantly adding new groups.

In addition, PgUGTs were analysed for subcellular localisation prediction, chromosomal distribution, collinearity, gene structure, conserved motifs, and cis-regulatory elements. Notably, only 32 tandem duplication gene pairs and 5 fragment duplication gene pairs were identified in 107 PgUGTs. A total of 110 UGT gene pairs in Epimedium pubescens Maxim were considered to originate from tandem duplication events [32], and 7 duplicate gene pairs were identified from the Punica granatum [33]. This suggests that gene duplication events probably play a less driving role in PgUGTs compared to other species, but tandem and segmental duplication plays a significant role in the expansion process of the PgUGT family.

Subcellular localisation predictions showed that 89.72% of PgUGTs were localised in the cytoplasm or chloroplasts, and cis-acting elements analyses indicate the most light-responsive elements in PgUGTs. Previously, Pgβ-glucosidase, which we identified and expressed in *P. grandiflorus* roots to remove glucose from PE to generate PD, showed an increasing trend after 6:00 p.m., peaking at 12:00 p.m., and its expression was enhanced in the dark environment [15]. These results suggest that PgUGT, which has the catalytic activity of glucosylation at the C3 position of *P. grandiflorus* saponins, may also be regulated by light. For this purpose, PgUGT and UGT with catalytic activity at the C3 position of *P. grandiflorus* saponins were compared by blast, and the PgUGTs with high similarity were selected for Rt-qpcr detection. The results show that most of these PgUGTs were highly expressed in leaves, which was similar to the PE content distribution status we previously studied. Thus, we selected PgUGT29 and PgUGT72, which had relatively high relative expression in roots, stems, and leaves, with the highest expression in leaves and the least in roots, for further study.

In vitro enzyme activity assays showed that PgUGT29 has the function of catalysing the production of PD3 from PD, whereas PgUGT72 has no similar function. With the rapid development of protein crystallisation and analysis techniques, the study of protein structure has become clearer and clearer, and the crystal structures of more and more GTs have been resolved [34,35,36]. According to the folding characteristics of the 3D structures of GTs, they can be classified into four types: GT-A, GT-B, GT-C, and GT-D. Chang et al. [37] showed as early as 2011 that all the reported crystal structures of plant UGTs showed GT-B folding. GTB proteins have distinct N-terminal and C-terminal structural domains, each of which contains a typical Rossmann fold. In this case, the glycosyl donor binding pocket is located in the C-terminal folding region, while the glycosyl acceptor binding pocket is located in the N-terminal folding region. As mentioned earlier, UGTs recognise and bind the UDP-glycosyl via the C-terminal PSPG motif, while the N-terminal structural domain is loosely bound to the glycoside, which may lead to a diversity of substrate structures. Through multiple sequence comparisons of PgUGT29 with PgUGT72 and PgGT1, combined with molecular docking of PgUGT29 and UDP-Glc, we identified that both PgUGT29 and PgGT1 share four conserved residues (T145, D392, Q393, and N396). These residues were found to form stable hydrogen bonds with UDP-Glc during the docking simulation. Based on these structural observations, we hypothesise that T145, D392, Q393, and N396 may constitute critical residues responsible for UDP-Glc recognition and binding in PgUGT29.

## 4. Materials and Methods

### 4.1. Plant Materials

The *P. grandiflorus* plant used in this study is the same material described by Su et al. (2021) [17]. Specimens of *P. grandiflorus* are preserved in the herbarium of Anhui University of Chinese Medicine (depository number 20200705). Seedlings were grown at 25 ± 2 °C during the day and 23 ± 2 °C at night in the herbal garden of Anhui University of Chinese Medicine, Anhui province, China. Each sample in this study had three independent biological duplicates, and the collected samples were immediately stored at −80 °C and used in the experiment within 12 h.

### 4.2. Genome-Wide Identification of PgUGTs and Construction of Phylogenetic Tree

The genome database, protein database, and related annotation files of *P. grandiflorus* were downloaded from the National Genome Data Centre (https://ngdc.cncb.ac.cn, accessed on 8 July 2024), ID: PRJCA003843 [38]. The hidden Markov model (HMM) files of UGT transcription factor-conserved domains (PF00201) [13] were downloaded from the Pfam database (http://pfam.xfam.org/, accessed on 9 July 2024), and HMM Search was performed on the *P. grandiflorus* protein data, with the E-value set to e^−10^, to obtain the PgUGTs. Then, in Bioedit software, PSPG boxes of these PgUGTs were queried and validated to obtain the final protein dataset [39]. Finally, the physicochemical properties of these protein sequences were evaluated with the ProtParam tool of ExPASy5 [40] and the protein subcellular localisations were predicted on the Busca online website (http://busca.biocomp.unibo.it/, accessed on 14 July 2024) [41]. In order to analyse the evolutionary relationships of UGT protein sequences, we selected eighteen *Arabidopsis thaliana* UGTs belonging to groups A-N, four *Zea mays* UGTs belonging to groups O, P, and Q, and two *Camellia sinensis* UGTs belonging to group R by reading the literature and looking it up on the NCBI (Supplementary Table S3). Subsequently, the sequence alignment of PgUGTs with these identified grouped UGTs was performed in MEGA 11 using the Clustal W method, and the neighbour-joining method was used to generate the evolutionary tree, with the bootstrap value set to 1000 and pairwise deletion of gaps and p-distance methods [42]. The evolutionary tree was landscaped at the itol online URL (https://itol.embl.de/, accessed on 19 July 2024) [43].

### 4.3. Chromosomal Localisation and Collinearity Analysis

The chromosomal positional information of *PgUGT* genes and chromosome lengths were obtained from the *P. grandiflorus* genome. According to the relevant literature [44], MG2C (http://mg2c.iask.in/mg2c_v2.1/, accessed on 21 July 2024) [45] and TB-tool software were used to perform chromosomal localisation and collinearity analysis.

### 4.4. Cis-Regulatory Elements Analysis

TBtools [46] was used to obtain a 2000 bp sequence upstream of the PgUGT from the start codon. The cis-regulatory elements of the promoter sequence were detected on the plant care website (https://bioinformatics.psb.ugent.be/webtools/plantcare/html/, accessed on 21 July 2024) [47], and the elements were later displayed using TBtoolsⅡ.

### 4.5. Motif, Domain, and Gene Structure Analysis

The conserved motifs of PgUGTs were analysed using the online website Meme (http://meme-suite.org/tools/meme, accessed on 22 July 2024) [48].The number of motifs was 10 and the width of motifs was 6–50. The domains of PgUGTs were extracted by the Batch CD-Search tool of NCBI (https://www.ncbi.nlm.nih.gov/Structure/bwrpsb/bwrpsb.cgi, accessed on 22 July 2024) [49] and displayed using the TBtoolsⅡ. The intron and exon regions of the PgUGT genes were analysed using the gene structure visualisation program of TBtools.

### 4.6. Total RNA Extraction, cDNA Synthesis, and RT-qPCR

Total RNA was extracted using the TransZol Kit (Transgen Biotech, Inc,. Beijing, China) according to the manufacturer’s instructions. FastKing RT Kit (Tiangen Biotech Co., Ltd., Beijing, China) reverse transcription reaction was used to synthesise cDNA. Primers were designed with Primer 5.0 software (Supplementary Table S4), and primer synthesis was performed by Shanghai Bioengineering. The 18srrna gene was used as the internal reference gene. The SYBR Green qPCR Master Mix kit (Vazyme Biotech Co., Ltd., Beijing, China) was used for RT-qPCR experiments. The amplification program consisted of a pre-denaturation at 95 °C for 15 min, with three steps to amplify 40 cycles: 95 °C for 10 s, 58 °C for 20 s, and 72 °C for 20 s. Data were calculated using the 2^−ΔΔCt^ method to determine the relative gene expression levels. One-way analysis of variance (ANOVA) was applied for data processing, and statistical differences were compared using *t*-tests based on IBM SPSS Statistics 23. Finally, visual analysis was performed using GraphPad Prism 8.0.1.

### 4.7. Heterologous Expression and Purification of PgUGTs in Escherichia coli BL21 (DE3)

The UGT genes were synthesised by PuJian AtaGenix (Wuhan) Co., Ltd. (Wuhan, China) and subcloned into the pET-28α-3C expression vector. The expression vector was transformed into *E. coli* DH5α competence cells by the freeze-thawing method, and positive transformants were screened for amplification and sequencing on LB plates containing kanamycin (final concentration 50 ug/mL). The constructed recombinant expression vectors were transformed into *E. coli* BL21 (DE3) strains for protein expression. The transformed BL2 1 (DE3) strain was cultured in LB solution containing 50 μg/mL kanamycin at 37 °C to achieve an OD6 0 0 of 0.6~0.8. After the addition of isopropyl-β-D-thiogalactopyranoside (IPTG) at a final concentration of 1 mM to the culture medium, the culture was induced at 37 °C at 200 r/min for 4 h. The bacterial fluid was collected into centrifuge tubes after the completion of the induction. After centrifugation at 10,000× *g* for 1 min, the supernatant was discarded; 1 mL of 1 × PBS was added to the precipitate to ultrasonically lyse the cells. Subsequently, the cells were centrifuged at 10,000× *g* for 1 min, and the supernatant was added to a pre-equilibrated Ni-NTA column (Qiagen, Hilden, Germany) and incubated for 30 min at 4 °C. The cells were eluted with 10 mL of purification buffer (1 × PBS pH 7.5, 8 M urea) and 5 mL of elution buffer (PBS pH 7.5, 300 mM Imidazole, 8 M urea). The collected protein solution was added to a dialysis bag and dialysed overnight using 50 mM Tris-HCl (pH 7.0) buffer. The purified protein concentration was measured using the Bradford method.

### 4.8. SDS-PAGE and Western Blot Analyses

The samples were added with 5 × reducing loading buffer, boiled for 10 min, and loaded onto a pre-run gel (5% stacking gel and 12% resolving gel) with an initial voltage of 120 V. The voltage was adjusted to 200 V until electrophoresis was completed when the run was under the stacking gel. After staining with 0.125% Kaomas Brilliant Blue G-250 (Bio-Rad, Richmond, CA, USA), the colour was decoloured and photographed using an HP Scanjet G4050 photo scanner (Beijing, China).

After gel-electrophoresis, the pre-run gel was transferred onto a polyvinylidene fluoride (PVDF) membrane. The membrane was incubated overnight with primary antibody (Anti His tag mouse monoclonal antibody, dilution 1:5000) and then incubated for 1 h with secondary antibody (HRP-conjugated Affinipure Goat Anti-rabbit IgG, dilution 1:10,000). Ultimately, imaging was performed in a gel imaging system ChemiDoc^TM^ XRS+ (Bio-Rad).

### 4.9. Enzymatic Assays of UGTs In Vitro

High-performance liquid chromatography (HPLC) analysis was performed using the Agilent 1260 Infinity II LC System (Agilent Technologies, Santa Clara, CA, USA). The chromatographic column was a Topsil C18 column (4.6 mm × 250 mm; Agilent, Santa Clara, CA, USA) with a column temperature of 35 °C. The gradient elution system consisted of 0.1% phosphoric acid in water (A) and acetonitrile (B). The gradient elution procedure was as follows: 0 min (12% B), 0–1 min (15% B), 1–4 min (20% B), 4–8 min (28% B), 8–17 min (31% B), 17–25 min (33% B), 25–36 min (38% B), 36–50 min (44% B). The flow rate was maintained at 1 mL/min.

The enzyme analysis reaction system for glycosyltransferases was performed with reference to Zhang et al. [13] with minor modifications. The reaction was carried out in a 100 μL system consisting of 50 mM Tris-HCl (PH7.0) buffer, 50 μL protein, 14 mM β-mercaptoethanol, 5 mM UDP-Glc, and 1 mM receptor substrate. The reaction was carried out overnight in 30 °C, terminated with cold methanol the next day, and analysed by HPLC.

### 4.10. Molecular Docking

The structure model of PgGT29 was constructed by Robetta [50]. We downloaded the Mol2 file for UDP-Glc at the TCMSP database. Molecule docking of PgGT29/UDP-Glc was carried out by Autodock [51]. Finally, PyMOL V3.0 was used for molecular docking visualisation [52].

## 5. Conclusions

This study systematically identified 107 PgUGTs from the *P. grandiflorus* genome and characterised their phylogenetic classification, gene duplication patterns, subcellular localisation, and cis-regulatory elements. Phylogenetic analysis categorised these PgUGTs into 17 distinct groups, highlighting evolutionary divergence from UGT families in other plant species. Notably, tandem and segmental duplication events may contribute modestly to PgUGT family expansion compared to other species. Subcellular localisation predictions revealed cytoplasmic and chloroplast targeting for most PgUGTs, while cis-regulatory element analysis underscored light responsiveness as a key regulatory feature. Functional characterisation of PgUGT29 demonstrated its ability to catalyse the glucosylation of PD to PD3, a critical step in saponin biosynthesis, whereas PgUGT72 lacked this activity. Structural insights from molecular docking identified T145, D392, Q393, and N396 as conserved residues essential for UDP-Glc recognition in PgUGT29, aligning with the canonical GT-B folding architecture of plant UGTs. These findings not only advance our understanding of the molecular basis for substrate specificity in PgUGTs but also provide a foundation for elucidating glycosylation mechanisms in *P. grandiflorus* saponin biosynthesis, with potential implications for metabolic engineering and natural product development.

## Data Availability

The original genome database, protein database, and related annotation files of *P. grandiflorus* are openly available in the National Genome Data Centre (https://ngdc.cncb.ac.cn), ID: PRJCA003843. The hidden Markov model (HMM) files of UGT transcription factor-conserved domains (PF00201) are openly available in the Pfam database (http://pfam.xfam.org/).

## Supplementary Materials

The following supporting information can be downloaded at: https://www.mdpi.com/article/10.3390/ijms26104832/s1.
