# Supplementary material for: Genome-Wide Identification of UGT Gene Family and Functional Analysis of PgUGT29 in Platycodon grandiflorus
Source: Int J Mol Sci. 2025 May 18;26(10):4832. doi: 10.3390/ijms26104832 (PMC12112217; doi:10.3390/ijms26104832)
Supplement: Supplementary file 1 [file ijms-26-04832-s001.zip › Supplementary Figures.pdf]

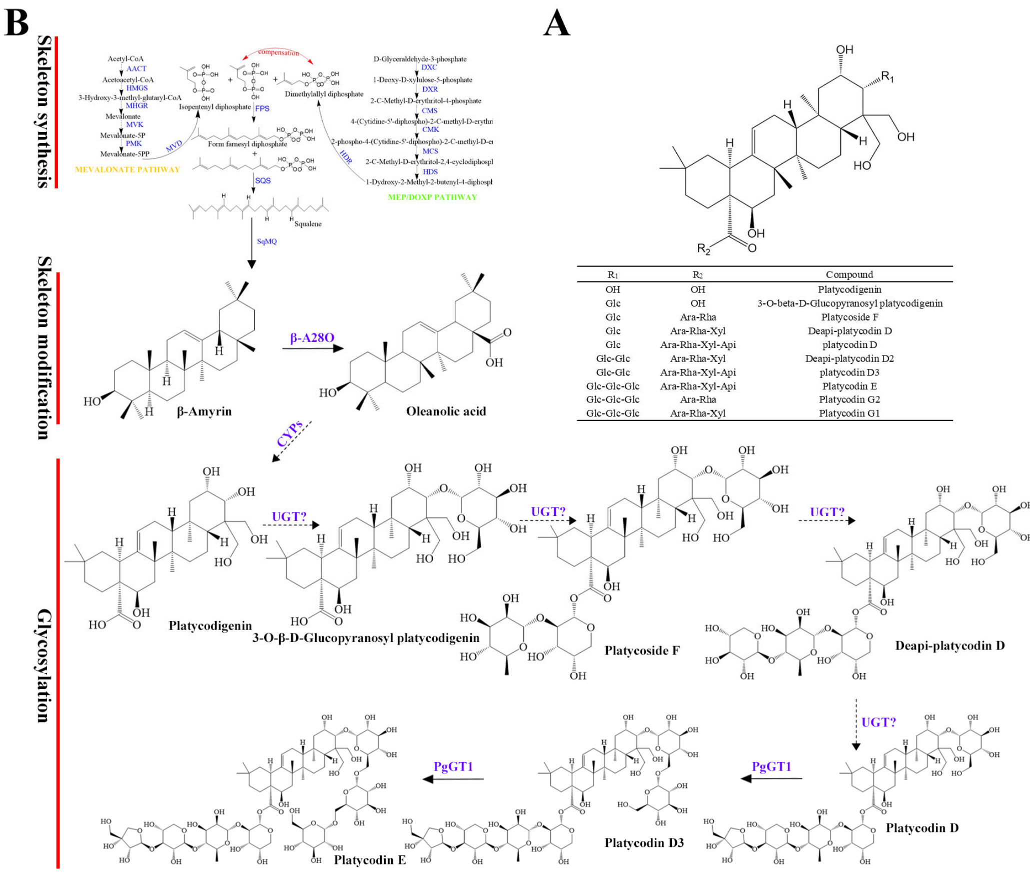

R1	R2	Compound
OH	OH	Platycodigenin
Glc	OH	3-O-beta-D-Glucopyranosyl platycodigenin
Glc	Ara-Rha	Platycoside F
Glc	Ara-Rha-Xyl	Deapi-platycodin D
Glc	Ara-Rha-Xyl-Api	platycodin D
Glc-Glc	Ara-Rha-Xyl	Deapi-platycodin D2
Glc-Glc	Ara-Rha-Xyl-Api	platycodin D3
Glc-Glc-Glc	Ara-Rha-Xyl-Api	Platycodin E
Glc-Glc-Glc	Ara-Rha	Platycodin G2
Glc-Glc-Glc	Ara-Rha-Xyl	Platycodin G1

Supplementary Figure S1 The platycodic acid saponins and putative synthetic pathway. (A) The platycodic acid saponins. (B) Putative synthetic pathway. Catalytic enzymes are in blue font and compounds are in black font. Solid lines indicate that the reaction process was determined, dashed lines indicate that the reaction was not validated or involved multiple steps.

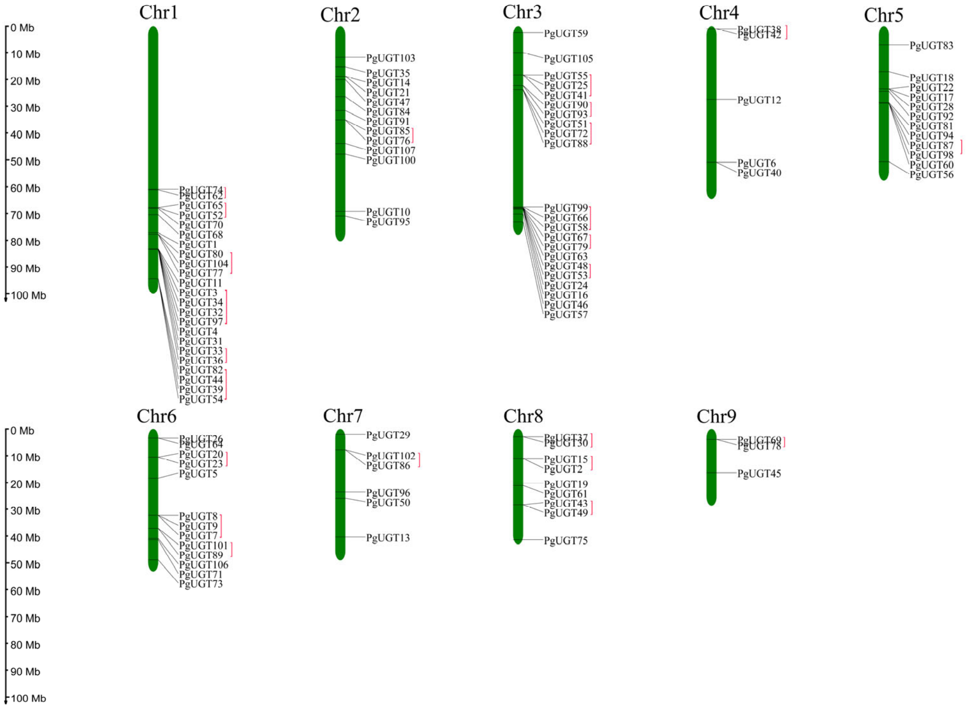

Supplementary Figure S2 Chromosomal location and tandem duplication event analyses of *PgUGT* genes. The tandem duplicated gene pairs are connected by red lines.

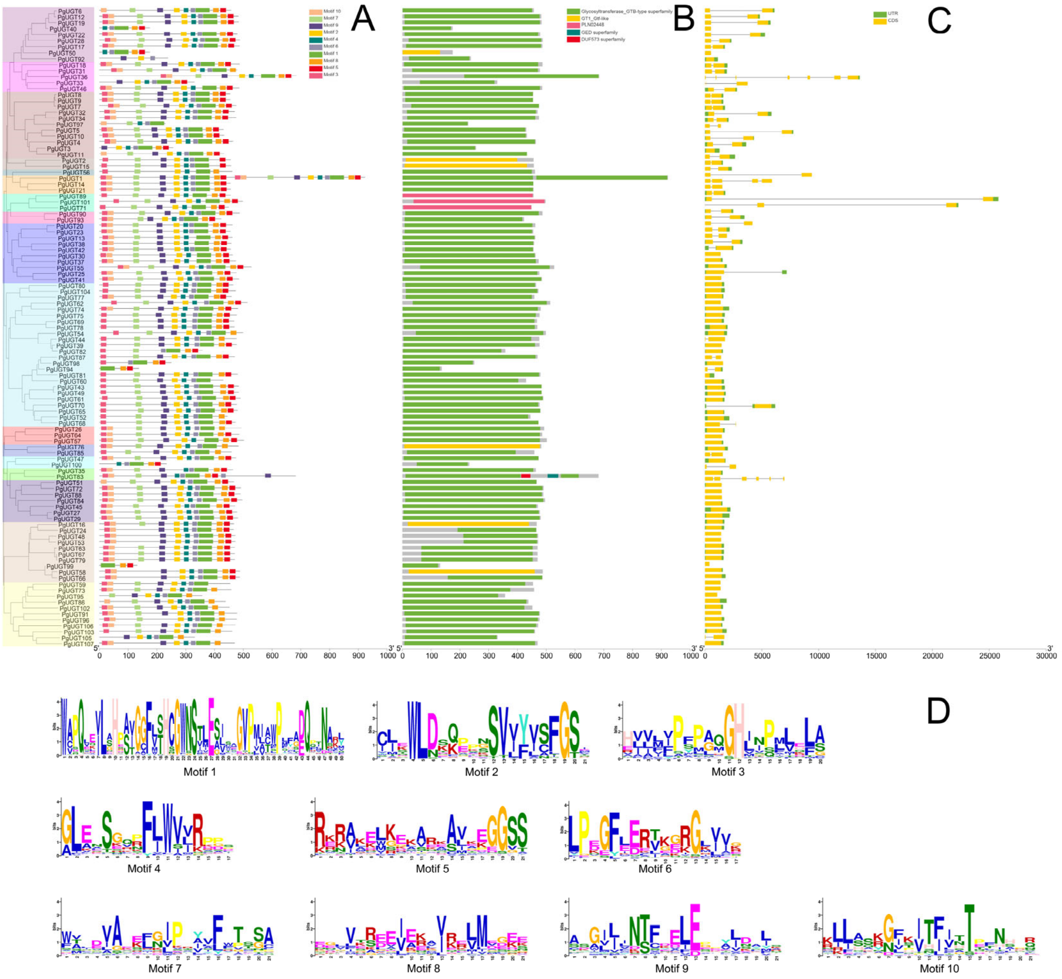

Supplementary Figure S3 Conserved motifs, conserved domains and gene structures analysis in PgUGTs. (A) The motif composition of PgUGT proteins. Numbers 1–10 of motifs are shown in different colored boxes. (B) gene domains of PgUGTs. 5 domains in 5 colored boxes. (C) Exon-intron structure of PgUGTs. Yellow boxes indicate exons, green boxes indicate UTR, black lines indicate introns. (D) Amino acid composition analysis of motif 1-10.

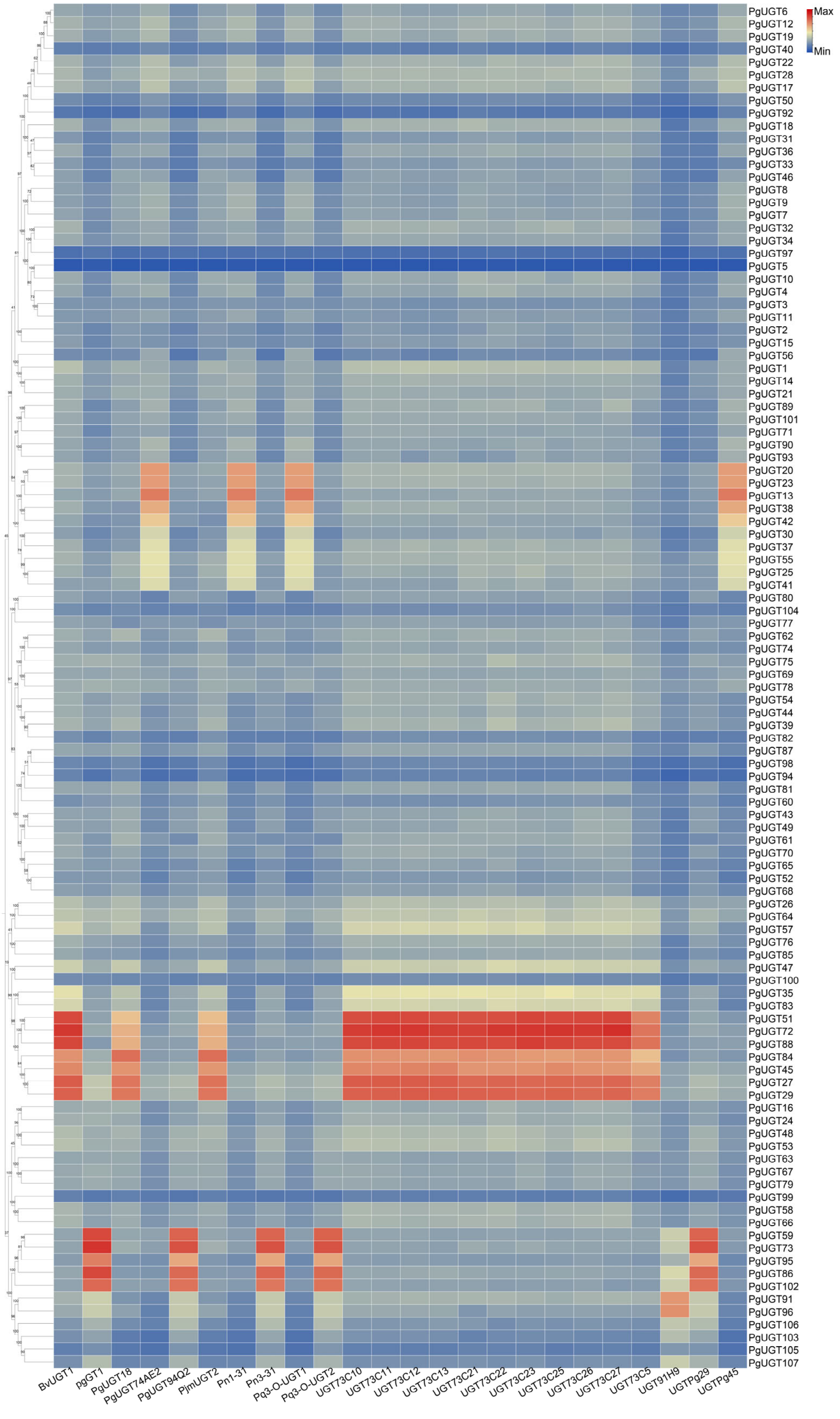

Supplementary Figure S4 Heat map of blast bit score between PgUGTs and 24 functionally determined UGT.
